# Low-concentration contrast abdominopelvic CT: A comparison with high-concentration contrast CT image quality

**DOI:** 10.1371/journal.pone.0338726

**Published:** 2026-01-05

**Authors:** Sungjin Yoon, Joo-Hwan Park, Sun Jin Sym, Dong Hyuk Yang, Ji Sung Lee, So Hyun Park

**Affiliations:** 1 Department of Radiology, Gil Medical Center, Gachon University College of Medicine, Incheon, Republic of Korea; 2 Department of Radiology, Incheon Sejong hospital, Incheon, Republic of Korea; 3 Division of Medical Oncology, Department of Internal Medicine, Gil Medical Center, Gachon University College of Medicine, Incheon, Republic of Korea; 4 Department of Clinical Epidemiology and Biostatistics, Asan Medical Center, Ulsan University College of Medicine, Seoul, Republic of Korea; 5 Department of Radiology, Seoul National University Bundang Hospital, Seoul National University College of Medicine, Seongnam, Republic of Korea; Coventry University, UNITED KINGDOM OF GREAT BRITAIN AND NORTHERN IRELAND

## Abstract

**Objective:**

This study aimed to compare abdominopelvic computed tomography (APCT) image quality using low and high concentrations of a contrast agent in patients with cancer via a noninferiority design.

**Methods:**

This study prospectively included 99 patients with malignancies who underwent dual-source APCT following injection of 1.5 mL/kg of iohexol 270 (low-concentration group). The control group included patients who were retrospectively matched 1:1 based on weight, body mass index, and sex. These patients (high-concentration group) received 1.1 or 1.2 mL/kg of iohexol 350, reconstructed at two tube voltages (80 kVp and 80/Sn150 kVp). The noninferiority margins were set at −0.21 for overall image quality. Two radiologists blindly and independently analyzed the subjective and objective image quality of matching focal lesions.

**Results:**

A total of 198 patients with 102 focal lesions (44 liver lesions, 58 renal lesions) were assessed. The total iodine amount was slightly higher in the low-concentration group than in the high-concentration group without significant differences (24580.0 ± 3745.6 vs. 24190.2 ± 3954.3 mg I/mL; *p* = 0.051). Overall image quality did not differ significantly between the groups (2.6 ± 0.5 vs. 2.6 ± 0.5 on 80 kVp; *p* = 0.484; 3.4 ± 0.5 vs. 3.4 ± 0.6 on 80/Sn 150 kVp, *p* = 0.891). Margin sharpness and conspicuity also showed no significant differences between the low- and high-concentration groups at 80 kVp (*p* = 0.890, 0.103) and 80/Sn 150 kVp images (*p* = 0.278, 0.369). Liver attenuation was slightly higher in the low-concentration than in the high-concentration group (80 kVp, 127.3 ± 16.8 vs. 122.4 ± 13.0, *p* = 0.013; 80 kVp/Sn 150 kVp, 108.2 ± 13.3 vs. 104.5 ± 10.9, *p* = 0.025). No major or minor adverse reactions occurred during or immediately after contrast agent injection in either group. Five patients in the low-concentration group experienced below-average vascular pain.

**Conclusions:**

The low-concentration group offered noninferior overall image quality compared to the high-concentration group among patients with cancer.

## Introduction

Computed tomography (CT) plays a crucial role in the management of patients with cancer by enabling accurate disease staging, guiding therapeutic strategies, assessing treatment responses, and detecting disease recurrence or metastasis. While iodinated contrast agents are essential for detecting and characterizing lesions in abdominal CT examinations, they are known to precipitate adverse reactions, such as contrast-induced acute kidney injury [1–3]. The contrast administration protocol, including the total dose, concentration, injection rate, and injection duration, can be modified to mitigate the risk of adverse reactions. However, these parameters substantially influence not only the incidence of adverse reactions [4–7] but also the degree of contrast enhancement in vascular and parenchymal structures on CT images [8–11]. Therefore, it is necessary to adjust them judiciously to avoid compromising diagnostic image quality.

Some studies have reported increased liver enhancement when using high concentrations of contrast agents [12,13]. However, the degree of contrast enhancement in CT images is complex and influenced by many factors beyond contrast concentration. These factors include other contrast administration parameters, timing of image acquisition, and the target organ. In abdominal evaluation of patients with cancer, portal venous phase CT images are commonly acquired, where the enhancement of the CT image correlates more strongly with total iodine delivery than with the concentration of the contrast agent [14–19]. If a low-concentration contrast agent can provide image quality comparable to that of a high-concentration contrast agent, the incidence of contrast-induced nephropathy can be mitigated due to the diminished viscosity of the low-concentration contrast agent [7].

The tube voltage employed by the CT scanner is another factor that enhances contrast. When utilizing low tube voltages, the X-ray energy approaches the K-absorption edge of iodine, resulting in an increased degree of enhancement in the reconstructed image [20]. Dual-source dual-energy CT technology facilitates the separation or merging of data acquired from two distinct X-ray tubes during image reconstruction, enabling inter-individual comparison of images acquired from a single patient. This scanning technique permits the simultaneous acquisition of both 80 kVp and 120 kVp image series within a single examination.

Therefore, this study aimed to compare the image quality of abdominopelvic CT examinations performed in patients with cancer using low and high concentrations of a contrast agent via a noninferiority design. Additionally, we aimed to evaluate the effects of both low and standard tube voltage levels on image quality. Finally, we sought to assess the comparative frequency of adverse events related to contrast agent administration between patient groups receiving low and high concentrations of a contrast agent.

## Materials and methods

### Study participants

This single-center, case-control study was registered with the domestic trial registry of the Republic of Korea (cris.nih.go.kr: KCT0008622) and was approved by the Institutional Review Board of our institute (IRB No. GDIRB2022−309) in accordance with the Declaration of Helsinki. We matched the case-control groups as follows: the case group included patients with malignancies at the oncology department who were prospectively enrolled after obtaining written informed consent, using a low concentration of a contrast agent (iohexol 270 mg I/mL) for abdominopelvic CT between December 2022 and August 2023. The control group comprised patients who were examined with a high concentration of the contrast agent (iohexol 350 mg I/mL) during the same period. They were retrospectively matched 1:1 with the case group based on weight, body mass index, and sex. Informed consent was waived for patients in the control group due to the retrospective nature. The control group data accessed in de-identified form for this study were obtained between December 2022 and July 2024.

To be included in the case group, patients needed to have a confirmed malignancy, be between 18 and 80 years, and provide informed consent for the use of a low-concentration contrast agent (Iobrix^®^ inj. 270; Taejoon Pharm Co., Ltd., Seoul, Korea) for abdominopelvic CT using a dual-energy CT scanner. Patients were excluded if they had renal impairment (serum creatinine > 1.4 mg/dL or glomerular filtration rate <45 mL/min/1.73 m^2^) confirmed by blood tests within the previous month, if their CT protocol needed to be changed during the examination, if there was no matching patient in the control group, or if they had contraindications to Iobrix (hypersensitivity to the drug, its components, or iodinated agents, including history of severe thyroid disorders). Of the 104 patients who provided informed consent, two were excluded due to changes in their CT examination protocol, two were excluded for medical reasons, and one was excluded due to the absence of a matching patient in the control group. Thus, 99 patients were enrolled in the study group for abdominopelvic CT using low concentrations of the contrast agent.

**Fig 1:**
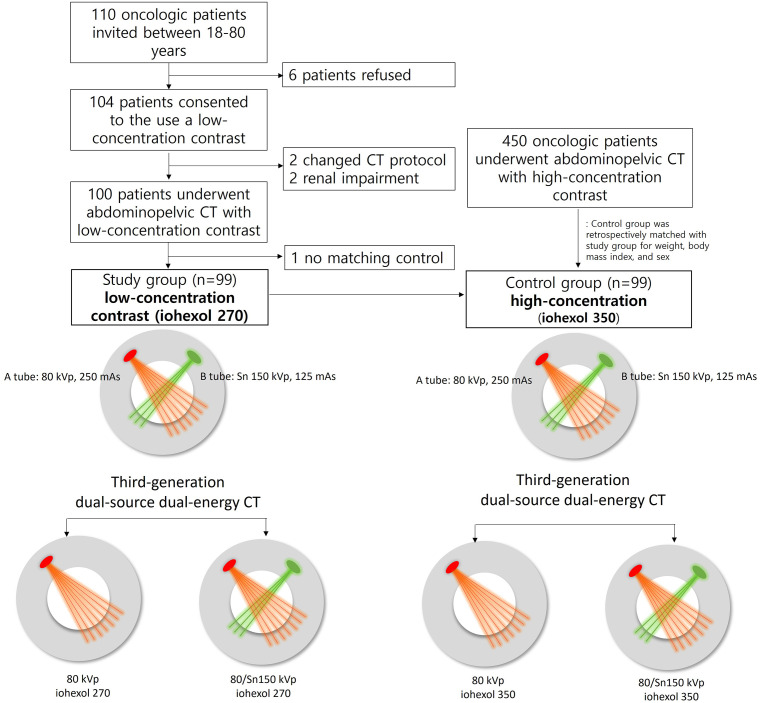
Study flowchart.

The control group, comprising patients with cancer who underwent abdominopelvic CT scans using a high concentration of the contrast agent (Iobrix^®^ inj. 350; Taejoon Pharm Co., Ltd., Seoul, Korea), was selected retrospectively in the same period. Precisely 450 oncologic patients underwent abdominopelvic CT with a high-concentration contrast agent during that period. Matching was performed 1:1 with the prospective case group based on weight (within ±8 kg), body mass index (±3 kg/m^2^), and sex. For the analysis of focal liver and kidney lesions, a study coordinator performed an additional 1:1 subgroup matching based on lesion size (±3 mm) in each organ between matched case-control patients. Finally, 99 patients were matched to the control group (Fig 1).

### CT image acquisition and reconstruction

Using dual-energy CT (SOMATOM Force; Siemens Healthcare, Forchheim, Germany), imaging was performed at 80 kVp in tube A (reference 360 mAs) and 150 kVp in tube B (reference 125 mAs). The combined 80 kVp/Sn 150 kVp image, which is similar to the conventional 120 kVp image, was evaluated for image quality. With a single scan using dual-energy CT, patients can acquire two images (i.e., low- and high-kVp CT images) without additional exposure for comparison.

In the low-concentration contrast CT scans of the prospective case group, portal venous phase images were acquired 70 s after contrast injection of iohexol (270 mg I/mL, 1.5 mL/kg, and 3 mL/s), with a maximum injection volume not exceeding 222 mL. In the high-concentration contrast CT of the control group, portal venous phase images were acquired 70 s after contrast injection of iohexol (350 mg I/mL, 1.1 or 1.2 mL/kg, and 3 mL/s), with a maximum injection volume not exceeding 150 mL. For patients who could not tolerate an injection rate of 3 mL/s due to poor vascular conditions, the injection rate was reduced to 2 mL/s. The total amount of iodine was expected to be the same in both groups.

### Qualitative image analysis

Two abdominal radiologists (S.Y. and S.H.P.) who were blinded to the clinical information independently reviewed the CT images. They used a 4-point scale to evaluate the following items: overall image quality (1: significantly poorer than average, 2: poorer than average, 3: average, 4: better than average), artifacts (1: strong artifacts, insufficient for diagnostic purposes, 2: severe artifacts causing uncertainty, 3: moderate artifacts with mild restricted assessment, 4: slight or minimal artifacts, allowing unrestricted diagnostic image evaluation), image noise (1: strong noise, insufficient for diagnostic purposes, 2: severe noise causing uncertainty, 3: moderate noise with mild restricted assessment, 4: slight or minimal noise with unrestricted diagnostic image evaluation), and contrast (1: significantly poor, 2: poorer than average, 3: fair, 4: good).

### Qualitative image analysis for focal liver and renal lesions

The reviewers assessed the sharpness and conspicuity of the matched lesions in the liver and kidneys. The conspicuity and margins of the lesions were evaluated using a 5-point scoring scale (1: significantly poor, 2: poorer than average, 3: fair, 4: good, and 5: excellent).

### Reference standard

Focal liver and renal lesions were selected by the study coordinator for subgroup analysis by 1:1 size matching (±3 mm). Lesion matching was performed in a blinded manner, and lesion size distributions were re-validated following the subgroup selection to minimize selection bias. Patients who underwent liver magnetic resonance imaging within 1 month, had pathologically confirmed lesions, or had stable lesions on imaging for 1 year were included in the analysis. Patients with >10 focal liver or renal lesions were excluded from the analysis.

### Quantitative image analysis

One blinded reader drew a circular region of interest (1–3 cm^2^) in the right hemiliver, portal vein, and subcutaneous fat layer on CT images acquired at 80 kVp and 80 kVp/Sn 150 kVp in the low- and high-concentration contrast agent groups. The average attenuation and image noise (in Hounsfield units) in the region of interest were measured using a picture archiving and communication system.

### Evaluation of adverse reaction

If any adverse reactions were observed after CT examinations, patients were treated following the standard management for contrast media reactions, and the details were documented in the electronic medical records. If vascular pain during contrast injection was extremely severe or perceived to differ from previous examinations, the physicians (S.J.S. and J.H.P.) documented the details in the electronic medical records.

### Statistical analysis

Sample size calculation was based on a margin of noninferiority for image quality score set at −0.21 [21–23]. Assuming a common standard deviation of 0.5, with a power of 80% and a 1-sided α-level of 0.025, we estimated that 90 patients in each group would be needed to demonstrate the noninferiority of the overall image quality of CT scans using the high- concentration contrast agent considering a 15% dropout rate,. Conditional logistic regression was used to compare the patient characteristics and qualitative data for the clustering of matched pairs. Quantitative data between the matched pairs were compared by linear regression using a generalized estimating equation method with robust standard errors. Statistical significance was set at a *p*-value < 0.05.

Interobserver agreement regarding subjective image quality was assessed using the intraclass correlation coefficient (ICC). The ICC values were interpreted as follows: poor, < 0.40; fair, 0.40–0.59; good, 0.60–0.74; excellent, 0.75–1.00 [24]. All statistical analyses were performed using statistical software (SPSS version 22.0; IBM, Armonk, NY, USA;; and PASS, version 15.0.7) and SAS version 9.4 (SAS Institute Inc., Cary, NC, USA).

## Results

### Patient characteristics

The clinical characteristics of the patients are summarized in Table 1. Of the 198 patients, 99 patients each were assigned to the low- and high-concentration contrast agent groups. Both groups had similar sex distributions (66 male and 33 female patients). No significant differences were found between the two groups in terms of weight (*p *= 0.422), body mass index (*p *= 0.161), asthma or allergies (*p *= 0.147), diabetes mellitus(*p *= 0.847), metformin use (*p *= 0.706), renal disease (*p *= 0.069), past renal surgery (*p *= 0.571), heart disease (*p *> 0.999), or hypertension (*p *= 0.260). The total amount of iodine administered was comparable between the groups (24580.0 ± 3745.6 vs. 24190.2 ± 3954.3 mg I/mL; *p* = 0.051). However, due to different injection volume settings (1.5 mL/kg vs. 1.1 mL/kg), the total contrast volume in the low-concentration contrast agent group (91.0 ± 13.9) was higher than that of the high-concentration contrast agent group (69.1 ± 11.3, *p* < 0.001, S2 Table).

**Table 1:** Patient characteristics and contrast agent.

Parameter	Total	Low- concentration contrast agent	High- concentration contrast agent	*p-*value†
**Patients**	198	99	99	
** Male**	126 (63.6)	63 (63.6)	63 (63.6)	N/A
** Age (years)**	66.2 ± 9.8	63.8 ± 8.4	68.5 ± 10.5	0.001
** Height (cm)**	163.2 ± 8.4	163.8 ± 8.0	162.6 ± 8.8	0.011
** Weight (kg)**	62.1 ± 9.8	62.3 ± 9.6	61.9 ± 10.0	0.422
** Body mass index (kg/m**^**2**^)	23.3 ± 3.2	23.2 ± 3.2	23.4 ± 3.2	0.161
** Asthma or allergies**	11 (5.6)	3 (3.0)	8 (8.1)	0.147
** Diabetes mellitus**	33 (16.7)	17 (17.2)	16 (16.2)	0.847
** Metformin medication**	7 (3.5)	3 (3.0)	4 (4.0)	0.706
** Medical renal disease**	8 (4.0)	1 (1.0)	7 (7.1)	0.069
** Past renal surgery**	3 (1.5)	1 (1.0)	2 (2.0)	0.571
** Heart disease**	16 (8.1)	8 (8.1)	8 (8.1)	>0.999
** Hypertension**	82 (41.4)	45 (45.5)	37 (37.4)	0.260
**Contrast parameters**				
**Iodine concentration (mg I/mL)** ^*^	270 or 350	270	350	N/A
**Total contrast volume (mL)**	80.1 ± 16.7	91.0 ± 13.9	69.1 ± 11.3	<0.0001
**Total iodine amount (mg)** ^*^	24385.1 ± 3846.6	24580.0 ± 3745.6	24190.2 ± 3954.3	0.051
**Focal lesions**				
** Focal liver lesions**	44	22	22	
** Size (mm)**	7.7 ± 3.9	7.5 ± 4.0	7.8 ± 3.8	0.731
** Focal renal lesions**	58	29	29	
** Size (mm)**	37.2 ± 7.0	35.8 ± 7.4	38.6 ± 6.3	0.119
** Focal lesions**	102	51	51	
** Size (mm)**	24.5 ± 15.8	23.6 ± 15.4	25.4 ± 16.3	0.567

Note: Data are presented as numbers (%), unless indicated otherwise.

Data are presented as mean ± standard deviation. N/A, not applicable

† *p*-value was determined by conditional logistic regression that accounted for the clustering of matched pairs. *p-value* < 0.05 was considered statistically significant.

* Iodine concentration (mg I/mL) denotes the concentration of iodine in the contrast medium, while the total iodine amount (mg) denotes the actual amount of iodine administered.

Heart diseases include coronary artery disease and heart failure.

For subgroup focal lesion analysis by 1:1 size matching (±3 mm) between the low- and high-concentration contrast agent groups, a total of 102 lesions were matched (focal liver lesions = 44, focal renal lesions = 58). No significant difference was observed in the mean size of the lesion (23.6 ± 15.4 vs. 25.4 ± 16.3, *p* = 0.567). Of the 44 patients with focal liver lesions, 22 were in the low-concentration group (cyst = 11, hemangioma = 4, metastasis = 6, calcification = 1) and 22 were in the high-concentration contrast agent group (cyst = 14, hemangioma = 2, metastasis = 4, calcification = 2). Of the 58 patients with renal focal lesions, 29 were in the low-concentration group (cyst = 29) and 29 to the high-concentration contrast agent group (cyst = 27, metastasis = 1, inflammation = 1).

### Subjective image quality between low- and high-concentration contrast agent groups

The overall image quality did not significantly differ between the two groups (2.6 ± 0.5 vs. 2.6 ± 0.5 on 80 kVp, *p* = 0.484; 3.4 ± 0.5 vs. 3.4 ± 0.6 on 80/Sn 150 kVp, *p* = 0.891) as shown in Table 2. While noise was better in the high-concentration contrast agent group than in the low-concentration contrast group for 80/Sn 150 kVp images (3.7 ± 0.4 vs. 3.6 ± 0.5, *p* = 0.034), no significant differences were observed in artifacts (*p* = 0.279 for 80 kVp, 0.355 for 80/Sn 150 kVp) or contrast (*p* = 0.622, 0.278) between the groups. Both radiologists evaluating the images showed good agreement on overall quality [ICC = 0.678 (low-concentration contrast), 0.647 (high-concentration contrast)], fair consistency in artifacts (0.525, 0.417), and good-to-excellent consistency in image noise (0.725, 0.837) and image contrast (0.715, 0.753). Interestingly, the 80/Sn 150 kVp images showed fewer artifacts, lower image noise, but weaker contrast compared to 80 kVp images in both concentration groups (*p *< 0.001, all).

**Table 2:** Per-patient subjective image quality of abdominopelvic CT images in low- and high-concentration contrast agent groups.

Parameter	Total	Low concentration	High concentration	*p-value†*	Difference (95% CI)
**80 kVp**					
** Image quality**	2.6 ± 0.5	2.6 ± 0.5	2.6 ± 0.5	0.484	−0.05 (−0.19 − 0.09)
** Artifact**	3.5 ± 0.5	3.4 ± 0.5	3.5 ± 0.6	0.279	−0.08 (−0.23 − 0.06)
** Noise**	3.0 ± 0.2	3.0 ± 0.2	3.0 ± 0.2	0.706	0.01 (−0.04 − 0.06)
** Contrast**	3.8 ± 0.4	3.8 ± 0.4	3.7 ± 0.4	0.622	0.03 (−0.09 − 0.15)
**80/Sn 150 kVp**					
** Image quality**	3.4 ± 0.6	3.4 ± 0.5	3.4 ± 0.6	0.891	−0.01 (−0.15 − 0.13)
** Artifact**	3.9 ± 0.3	3.9 ± 0.3	3.9 ± 0.4	0.355	0.04 (−0.04 − 0.12)
** Noise**	3.7 ± 0.5	3.6 ± 0.5	3.7 ± 0.4	0.034	−0.14 (−0.27 − −0.02)
** Contrast**	3.0 ± 0.4	3.0 ± 0.5	2.9 ± 0.3	0.278	0.07 (−0.06 − 0.20)

Data are presented as mean ± standard deviation. CI, confidence interval.

† *p*-value was determined by conditional logistic regression that accounted for the clustering of matched pairs. *p-*value< 0.05 was considered statistically significant.

### Subjective image quality of focal lesions in low- and high-concentration contrast agent groups

**Fig 2:**
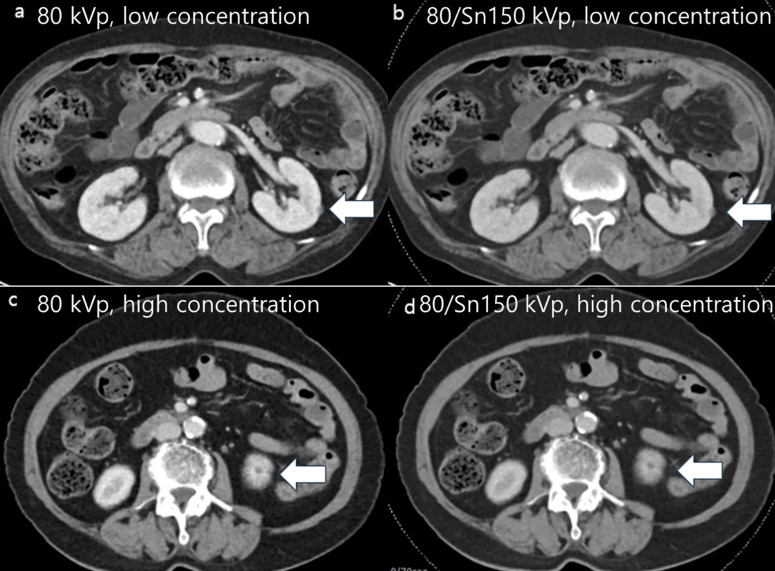
Abdominopelvic computed tomography (CT) images of patients with bladder and breast cancers. CT images of a patient with bladder cancer (76-year-old, female; weight, 59.3 kg; body mass index, 25.0 kg/m^2^; a-b) and a patient with breast cancer (80-year-old, female; weight, 55 kg; body mass index, 25.8 kg/m^2^; c-d) with matching cases. **(a-b)** CT images show a left renal cyst (5 mm in size) using a low concentration of contrast agent and (c-d) a left renal cyst (4 mm in size) using a high concentration of contrast agent. The overall image quality score was 2 for the 80 kVp CT image **(a)**, 3 for the 80/Sn 150 kVp image **(b)**, 2 for the 80 kVp CT image **(c)**, and 3 for the 80/Sn 150 kVp image **(d)**. The renal lesion conspicuity and margin sharpness were similar, showing a score of 3 for conspicuity in all images **(a-d)**, a score of 3 for margin sharpness in images (a) and **(c)**, and a score of 4 in images (b) and **(d)**.

**Fig 3:**
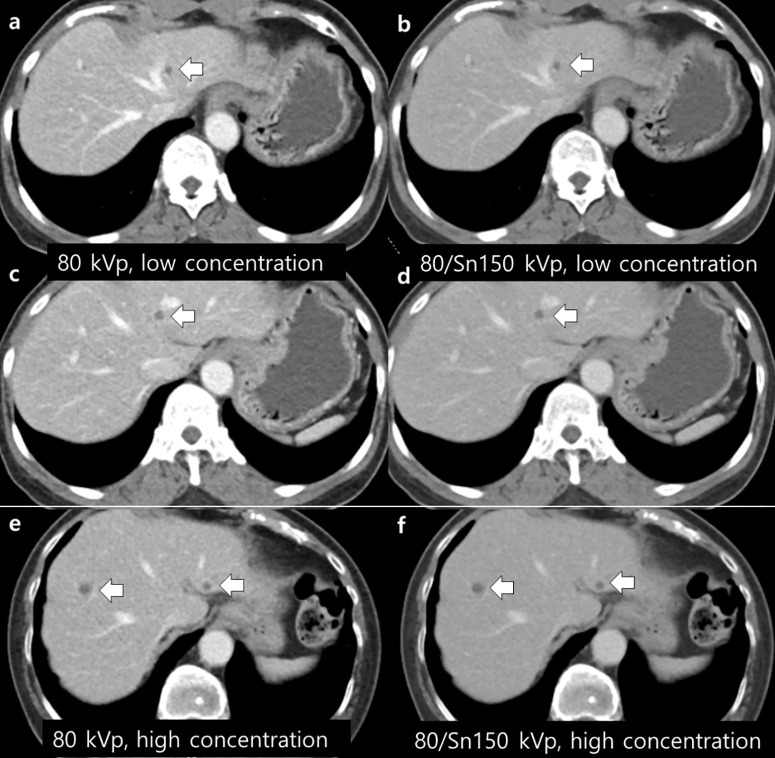
Abdominopelvic computed tomography (CT) images of two patients with breast cancers. CT images of two patients with breast cancer (64-year-old, female; weight, 57.0 kg; body mass index, 21.7 kg/m^2^; a-b) vs (80-year-old, female; weight, 52 kg; body mass index, 23.1 kg/m^2^; c-d) with matching cases. **(a-d)** CT images show two hepatic cysts (arrows, 5 and 6 mm in size, respectively) using a low concentration of the contrast agent, and (e-f) two hepatic cysts (arrows, 5 and 7 mm in size, respectively) using a high concentration of the contrast agent. The overall image quality was scored 3 for the 80 kVp CT image **(a and c)**, 4 for the 80/Sn 150 kVp image **(b and d)**, 3 for the 80 kVp CT image **(e)**, and 3 for the 80/Sn 150 kVp image **(f)**. The conspicuity and margin sharpness of the hepatic lesions were similar, with a score of 4 for both parameters in all images.

For the focal lesion subgroup analysis, 102 lesions (focal liver lesions = 44, focal renal lesions = 58) were assessed for margin sharpness and conspicuity in both low- and high-concentration contrast agent groups. In 80 kVp images, no significant difference was observed between the low- and high-concentration contrast agent groups for margin sharpness (3.9 ± 0.9 vs. 3.9 ± 1.0, *p* = 0.890) and conspicuity (3.9 ± 1.0 vs. 4.2 ± 0.8, *p* = 0.103). In 80 kVp/Sn 150 kVp images, no significant difference was observed between low- and high-concentration contrast agent groups for margin sharpness (3.7 ± 1.0 vs. 3.9 ± 1.0, *p* = 0.278) and conspicuity (3.5 ± 1.0 vs. 3.7 ± 0.8, *p* = 0.369). The details are presented in Table 3 and Figs 2–3.

**Table 3:** Per-lesion subjective image quality of focal liver and renal lesions in low-concentration and high-concentration contrast agent groups.

Organ	Low concentration	High concentration	*P-*value†
**80 kVp**			
** Liver lesions**			
** Margin**	3.4 ± 0.9	3.6 ± 1.0	0.300
** Conspicuity**	3.5 ± 0.9	3.9 ± 0.9	0.074
** Kidney lesions**			
** Margin**	4.2 ± 0.9	4.1 ± 1.0	0.664
** Conspicuity**	4.2 ± 0.9	4.4 ± 0.6	0.427
** Total**			
** Margin**	3.9 ± 0.9	3.9 ± 1.0	0.890
** Conspicuity**	3.9 ± 1.0	4.2 ± 0.8	0.103
**80/Sn 150 kVp**			
** Liver lesions**			
** Margin**	3.3 ± 0.9	3.6 ± 1.0	0.067
** Conspicuity**	3.1 ± 1.0	3.6 ± 1.0	0.028
** Kidney lesions**			
** Margin**	4.1 ± 0.9	4.1 ± 0.9	0.894
** Conspicuity**	3.9 ± 1.0	3.8 ± 0.6	0.571
** Total**			
** Margin**	3.7 ± 1.0	3.9 ± 1.0	0.278
** Conspicuity**	3.5 ± 1.0	3.7 ± 0.8	0.369

* Data are presented as mean ± standard deviation.

† *p*-value was determined by linear regression using the generalized estimating equation method with robust standard errors that accounted for the clustering of matched pairs. *p-*value < 0.05 was considered statistically significant.

### Objective image quality between low- and high-concentration contrast agent groups

The portal vein attenuation was significantly higher in the low-concentration contrast agent group than in the high-concentration contrast agent group (80 kVp images, 214.2 ± 24.8 vs. 193.8 ± 20.7, *p* < 0.001; 80 kVp/Sn 150 kVp images, 163.4 ± 17.6 vs. 152.6 ± 14.8, *p* < 0.001). The liver attenuation was higher in the low-concentration contrast agent group than in the high-concentration contrast agent group (80 kVp images, 127.3 ± 16.8 vs. 122.4 ± 13.0, *p* = 0.013; 80 kVp/Sn 150 kVp images, 108.2 ± 13.3 vs. 104.5 ± 10.9, *p* = 0.025). The liver noise was higher in the low-concentration contrast agent group than in the high-concentration contrast agent group (80 kVp images, 12.0 ± 1.7 vs. 11.3 ± 2.2, *p* = 0.012; 80 kVp/Sn 150 kVp images, 8.6 ± 1.3 vs. 7.8 ± 1.5, *p* = 0.001). The details are summarized in Table 4.

**Table 4:** Per-patient objective image quality in low-concentration and high-concentration contrast agent groups.

	Low concentration	High concentration	*p-*value†	Difference (95% CI)
**80 kVp**				
** Liver attenuation**	127.3 ± 16.8	122.4 ± 13.0	0.013	4.9 (1.0 − 8.8)
** Liver noise**	12.0 ± 1.7	11.3 ± 2.2	0.012	0.7 (0.2 − 1.3)
** Liver SNR**	10.8 ± 2.0	11.3 ± 2.5	0.159	−0.5 (−1.1 − 0.2)
** PV attenuation**	214.2 ± 24.8	193.8 ± 20.7	<0.0001	20.3 (14.2 − 26.5)
** PV noise**	13.4 ± 2.6	12.7 ± 4.2	0.130	0.7 (−0.2 − 1.7)
** Liver to PV CNR**	8.7 ± 3.0	7.3 ± 2.5	0.0003	1.3 (0.6 − 2.0)
** SQ attenuation**	−108.7 ± 20.1	−113.4 ± 35.0	0.224	4.7 (−2.9 − 12.3)
** SQ noise**	10.5 ± 2.0	11.6 ± 14.0	0.446	−1.1 (−3.9 − 1.7)
**80/Sn 150 kVp**				
** Liver attenuation**	108.2 ± 13.3	104.5 ± 10.9	0.025	3.7 (0.5 − 6.9)
** Liver noise**	8.6 ± 1.3	7.8 ± 1.5	0.001	0.8 (0.4 − 1.2)
** Liver SNR**	12.9 ± 2.6	13.9 ± 3.5	0.023	−1.0 (−2.0 − −0.1)
** PV attenuation**	163.4 ± 17.6	152.6 ± 14.8	<0.0001	10.7 (6.5 − 14.9)
** PV noise**	9.6 ± 2.0	9.3 ± 2.4	0.387	0.3 (−0.3 − 0.9)
** Liver to PV CNR**	7.2 ± 2.7	6.6 ± 2.5	0.081	0.6 (−0.1 − 1.3)
** SQ attenuation**	−97.3 ± 17.7	−99.1 ± 32.5	0.556	1.8 (−4.2 − 7.7)
** SQ noise**	8.2 ± 2.4	7.7 ± 1.9	0.068	0.6 (−0.04 − 1.2)

Data are presented as mean ± standard deviation. PV, portal vein; SQ, subcutaneous fat layer; CI, confidence interval

† *p*-value was determined by linear regression using the generalized estimating equation method with robust standard errors that accounted for the clustering of matched pairs. *p-*value < 0.05 was considered statistically significant

### Radiation dose of CT scans in low- and high-concentration contrast agent groups

The total dose length product was 138.7 ± 42.5 (low- vs. high-concentration contrast agent group; 135.5 ± 36.7 vs. 141.9 ± 47.5, *p* = 0.063) at 80 kVp and 255.3 ± 71.0 (250.0 ± 62.1 vs. 260.5 ± 78.9, *p* = 0.063) at 80 kVp/Sn 150 kVp. The data are summarized in Table 5. The total effective dose was 1.9 ± 0.6 (low- vs. high-concentration contrast agent group; 1.9 ± 0.6 vs. 1.9 ± 0.7, *p* = 0.091) at 80 kVp and 3.5 ± 1.1 (3.4 ± 1.0 vs. 3.6 ± 1.1, *p* = 0.092) at 80 kVp/Sn 150 kVp. The dose-length product and effective dose in the two groups (low- and high-concentration contrast agent group) for the 80 kVp (*p* = 0.063 for both) and 80 kVp/Sn 150 kVp scans (*p* = 0.091 and 0.092) were not significantly different.

**Table 5:** Radiation dose of CT scans in low- and high-concentration contrast agent groups.

Parameter	Total	Low concentration	High concentration	*p-*value†
**80 kVp**				
** CTDI**_**vol**_	2.5 ± 0.7	2.4 ± 0.6	2.6 ± 0.7	0.046
** DLP**	138.7 ± 42.5	135.5 ± 36.7	141.9 ± 47.5	0.063
** Effective dose**	1.9 ± 0.6	1.9 ± 0.6	1.9 ± 0.7	0.091
**80 kVp/Sn 150 kVp**				
** CTDI**_**vol**_	4.6 ± 1.1	4.5 ± 1.0	4.7 ± 1.2	0.041
** DLP**	255.3 ± 71.0	250.0 ± 62.1	260.5 ± 78.9	0.063
** Effective dose**	3.5 ± 1.1	3.4 ± 1.0	3.6 ± 1.1	0.092

* Data are presented as mean ± standard deviation. *p-*value < 0.05 was considered statistically significant.

### Adverse reactions

No major or minor adverse reactions occurred during or immediately after injection of the contrast agent in either group. Four patients (two each in the low- and high-concentration contrast agent groups) had poor vascular conditions, and the injection rate was reduced to 2 mL/s using a 20-gauge needle. Five patients in the low-concentration group experienced less vascular pain. There was no severe vascular pain during contrast medium injection in either group.

## Discussion

This study compared the image quality of abdominopelvic CT scans obtained using low and high concentrations of a contrast agent in patients with cancer. The comparative analysis revealed no statistically significant differences between the low- and high-concentration contrast agent groups in terms of overall image quality, artifacts, subjective contrast, margin sharpness, or conspicuity of focal lesions. Regarding the degree of enhancement, the low-concentration group showed slightly higher Hounsfield units in both the liver parenchyma and portal vein than the high-concentration contrast agent group. This difference is likely due to a slightly higher total iodine amount in the low-concentration contrast agent group than in the high-concentration contrast agent group, although the difference was not statistically significant. Overall, objective and subjective image quality assessments yielded similar results between the two groups. Additionally, there were no significant differences in the conspicuity and margins of size-matched focal lesions in the liver and kidneys between the low- and high-concentration contrast agent groups.

The influence of the contrast agent concentration was consistently observed at both 80 kVp and 80/Sn 150 kVp image acquisition protocols. We also evaluated the quality of abdominopelvic CT images acquired with low-kVp (80 kVp) compared to blended images generated by dual-energy CT (80/Sn 150 kVp), which mimic the use of high-kVp CT images. When using the same contrast agent concentration, 80 kVp scans offered superior contrast compared to 80/Sn 150 kVp. However, 80 kVp scans were inferior in terms of overall image quality, artifacts, and noise. Kim et al. demonstrated that low-tube-voltage CT using a low concentration contrast agent and iterative reconstruction yielded an image quality comparable to that of conventional images [25]. You et al. also reported that a dual-energy CT (80/Sn 150 kVp) blended image with a 0.6 blending factor resulted in the highest subjective image quality in the liver and was generally preferred among various CT images [26]. In our study, since the same reconstruction algorithm was used across protocols, the 80 kVp scans exhibited higher noise levels than the 80/Sn 150 kVp scans. Consequently, despite the superior image contrast at 80 kVp, the overall image quality for abdominopelvic CT scans was lower than at 80/Sn 150 kVp.

The optimal contrast agent injection protocol varies depending on the target organ and disease being evaluated [27]. For instance, in CT surveillance for hepatocellular carcinoma, the arterial phase is crucial for identifying hypervascular tumors, necessitating rapid administration of moderate-concentration contrast agents [28]. Additionally, using high concentrations of contrast agents during the arterial phase results in superior pancreatic enhancement [29]. Enhancement of the aorta and target organs during the arterial phase has been reported to correlate with the iodine delivery rate [30]. In patients with cancer, the portal phase plays a pivotal role in disease evaluation. Studies have shown that when the total iodine dose is held constant, the concentration of the contrast agent does not significantly affect the enhancement of vessels and the liver during the portal phase [14–19]. However, other studies have documented increased hepatic parenchymal enhancement with the administration of high concentrations of contrast agents [12,13] or impaired detectability of focal liver lesions when low concentrations of contrast agents are used [31]. These discrepancies across studies likely arise from variations in other injection parameters and tumor characteristics. In our study, although contrast enhancement was slightly higher in the low-concentration contrast agent group, the difference was not substantial. This finding is consistent with previous studies suggesting that contrast concentration is not significantly associated with image contrast.

Low concentrations of contrast agents offer advantages due to their lower viscosity and osmolarity, which translates to a reduced risk of contrast-induced acute kidney injury compared to high concentrations of contrast agents [32,33]. Several studies have compared CT image quality using low- and high-concentration contrast agents for various clinical applications. Meng et al. reported that a low concentration of contrast agent (270 mg I/mL) yielded image quality and diagnostic accuracy comparable to those when a high concentration of contrast agent (350 mg I/mL) was used for chest evaluation [34]. Zhou et al. reported no difference in CT image quality of the renal artery and vein when using contrast agents at concentrations of 270 and 320 mg I/mL [35]. Kim et al. demonstrated that iohexol 240 was non-inferior to iohexol 350 for evaluating the urinary tract in CT urography [25]. In this study, iohexol 270 was used to perform an overall evaluation of the abdomen and pelvis in patients with cancer and showed image quality comparable to that of iohexol 350. This demonstrates the feasibility of using iohexol 270 in abdominopelvic CT. Although no major or minor adverse reactions to the contrast agent were observed in either group, the small number of study patients may have hampered our ability to determine differences in the frequency of adverse reactions based on the concentration of the contrast agent. Interestingly, five patients in the low-concentration contrast group subjectively reported less vascular pain compared to past experiences, whereas none reported increased vascular pain in either group. This suggests a potential advantage of using low-concentration contrast agents, although the difference was small.

In our study, the patients in the low-concentration group received a larger total contrast volume and consequently had a longer injection duration than those in the high-concentration group while matching the total iodine amount between the two groups. These differences in injection parameters may influence the temporal characteristics of aortic and tissue contrast enhancement. Bae et al. reported that liver enhancement demonstrated similar or slightly higher attenuation with low-concentration contrast media (e.g., 300 mgI/mL) compared with high-concentration media (e.g., 400 mgI/mL) in the portal venous phase, based on simulated contrast enhancement curves generated with a fixed iodine mass and a fixed injection rate [36]. A longer injection with a larger volume can broaden the bolus profile, potentially lowering the peak enhancement but prolonging enhancement over time in the abdominal aorta [36]. Such a bolus shape may contribute to more uniform parenchymal enhancement during the portal venous phase, particularly in organs such as the liver and kidneys, and may be advantageous for oncology patients who undergo single-phase APCT in the portal phase, while potentially reducing the conspicuity of hypervascular lesions during the arterial phase. However, we did not investigate various patient factors such as the presence of hepatic disease and cardiac output. Additionally, the inability to standardize the injection rate in some patients and the use of a single-phase protocol prevented us from constructing attenuation curves for comparison between the two groups. Thus, future prospective studies employing multiphase acquisitions are required to enable a more precise evaluation of these temporal enhancement differences.

In this study, no significant difference in the total radiation dose was observed based on the concentrations of the contrast agent. Although iodinated contrast agents increase radiation dose due to photoelectric absorption [37], the variation in iodine content resulting from the different concentrations used was not substantial enough to elicit a detectable difference in radiation exposure.

This study had several limitations. First, the control group using a high concentration of the contrast agent was selected retrospectively with 1:1 matching, precluding a fully prospective study design. Second, although we aimed for equivalent total iodine amounts in the low- and high-concentration contrast agent groups, a slight discrepancy was observed in the actual amounts of total iodine administered. However, this difference was minimal (within 1%) and not statistically significant. Third, vascular pain occurring during the examination was not assessed using a standardized numeric pain rating scale. Our study reviewed the oncologists’ documentation in the electronic medical records. Future studies evaluating patients’ vascular pain with a scoring system and demonstrating its lower frequency in the low-concentration group would further strengthen the evidence supporting the use of low-concentration contrast media. Fourth, although the low- and high-concentration groups were matched for weight, body mass index, and sex, differences in height and age were observed between the two groups. However, as several studies [38–40] have reported that body weight is a significant factor influencing contrast enhancement, whereas age, height, and sex exert minimal effect, we believe that these differences are unlikely to have significantly affected our results. Finally, we could not evaluate diagnostic sensitivity/specificity for malignancy detection, but only assessed the image quality of CT scans and focal lesions in the liver, which represent a common metastatic site in patients with cancer. Future studies should assess the detectability of unknown or subtle lesions using reader studies with ground truth.

In conclusion, for follow-up examinations of patients with cancer, abdominopelvic CT utilizing a low concentration of a contrast agent with weight-based total iodine dose equivalence offers image quality comparable to scans acquired with a high concentration of the contrast agent.

## Supporting information

S1 Table Patient characteristics. (DOCX)

S2 Table Injection protocol parameters for low- and high-concentration groups. (DOCX)

S3 File STROBE Statement. (DOCX)
