# Supplementary material for: Low-concentration contrast abdominopelvic CT: A comparison with high-concentration contrast CT image quality
Source: PLoS One. 2026 Jan 5;21(1):e0338726. doi: 10.1371/journal.pone.0338726 (PMC12768260; doi:10.1371/journal.pone.0338726)
Supplement: S1 Table — (DOCX) [file pone.0338726.s001.docx]

S 1 table. Patient characteristics

| Primary malignancy | Low concentration  (n = 99) | High concentration  (n = 99) |
| --- | --- | --- |
| Colorectal cancer | 37 | 16 |
| Gastric cancer | 16 | 6 |
| Hepato-biliary cancer | 10 | 1 |
| Pancreatic cancer | 10 | 1 |
| Lung cancer | 8 | 31 |
| Breast cancer | 4 | 6 |
| Bladder cancer | 3 | 18 |
| Prostate cancer | 3 | 6 |
| Other malignancies | 8 | 14 |
